# Gastric metastases originating from occult breast lobular carcinoma: diagnostic and therapeutic problems

**DOI:** 10.1186/1477-7819-6-78

**Published:** 2008-07-25

**Authors:** Antonio Ciulla, Gioacchino Castronovo, Giovanni Tomasello, Alfonso Maurizio Maiorana, Leila Russo, Elio Daniele, Gaspare Genova

**Affiliations:** 1 Department of Oncology, Section of General Surgery, School of Medicine, University of Palermo, Italy; 2 Institute of Pathology, School of Medicine, University of Palermo, Italy

## Abstract

**Background:**

Breast cancer is the most frequent malignant tumour to metastasize into the gastrointestinal tract in female and is second only to malignant melanoma. Nevertheless gastrointestinal metastases arising from breast cancer are quite rare. The upper gastrointestinal tract is more frequently involved and lobular infiltrating carcinoma has a greater predilection compared to the ductal type.

**Case presentation:**

The authors describe the case of a 70 years old woman with a preoperative diagnosis of gastric undifferentiated medullary – type carcinoma, which was the first manifestation of an occult breast carcinoma. The primary site of carcinoma was identified with the use of a panel of selected immunohistochemical markers.

**Conclusion:**

Our goal in this case report is to increase the awareness of surgeons and clinicians to rule out the possibility of mammary origin in circumstance of gastric cancer occurring in female, even in patients without a previous or concurrent history of breast carcinoma. Although not a particularly common event, it is, nevertheless, reported in the literature. The differentiation between primary gastric carcinoma and metastatic breast carcinoma is essential for planning the correct therapeutic approach, in order to avoid the patient unnecessary surgery.

## Background

Breast cancer is the most frequent malignant tumour among women. Although breast carcinoma is after malignant melanoma the most commont primary tumour metastasizing to the gastrointestinal tract, mainly the stomach [1-4], such metastases occur only in 4–18% of patients [4].

Gastric metastases have been recognised in 6% of patients with disseminated breast cancer [1] and moreover the stomach may be the initial site of presentation [5,6]. Mammary malignant tumours show a distinctive systemic metastatic pattern. Ductal breast carcinoma is complicated by hepatic, lung and brain metastases, while upper gastrointestinal tract metastases are more often linked to lobular carcinoma [3,6,7].

The Authors describe the case of a 70-year-old woman with a pre-operative diagnosis of gastric undifferentiated medullary-type carcinoma, which was the first manifestation of an "occult" breast carcinoma.

## Case presentation

A 70-year-old apparently healthy woman with no obvious clinical history was admitted to medical examination in other Hospital. She had past history of generic dyspeptic symptoms, such as nausea and epigastric pain for last 10 years, in the last three months she had reported frequent episodes of vomiting and a weight loss of 8 Kg. Therefore she underwent an esophagogastroduodenoscopy, which demonstrated a widely hyperaemic gastric mucosa, with a nodular appearance of the fundus and corpus and antral hypertrophic plicae. The pylorus and duodenum looked quite normal. Several superficial biopsies of the gastric corpus were performed and in that contest the histology in association with a routined immunohistochemical analysis of the specimens took to the diagnosis of an "undifferentiated medullary type gastric carcinoma with focal neuroendocrine differentiation".

CT scan did not reveal any abdominal or nodal metastases. With evidence of absence of disease elsewhere, the patient underwent a total gastrectomy with lymphoadenectomy R1 and a mechanical T-L esophago-jejunostomy with a Roux loop technique.

**Figure 1:**
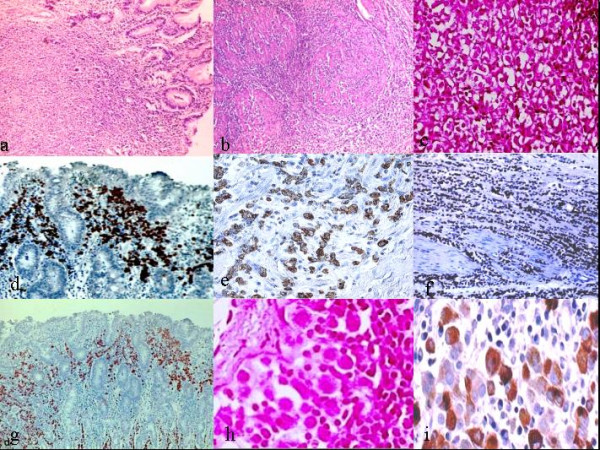
**Photomicrographs of stomach**. a)Small monotonous cells arranged in single elements crowed in mucosal layer (Haematoxylin-eosin original magnification 10×). b) Lenities plastic-like invasion of muscular layers (H&E original magnification 20×). c) Neoplastic cells with signet ring-like appearance: presence of an admixture of signet ring cells with single sharply circumscribed vacuoles and multivacuolated forms (hematoxylin-eosin, original magnification 40×). d-e) Neoplastic cells show a strong expression for cytokeratin 7 (original magnification 20×; 40×). f-g) Tumors cells show diffuse and strong nuclear positivity for oestrogenic receptors (original magnification 10×; 20×). h) Focus of Neoplatic cells. i) Cytoplasmatic positivity for gross cystic disease fluid protein 15.

Macroscopically the gastric mucosa of the fundus and corpus looked thinner than normal, with multiple brownish elevations, 18/18 perigastric lymph nodes resected were metastatic. Histological sections of the stump were stained with Hematoxylin-eosin. Immunohistochemistry using the strepavidin-avidina-biotina technique, was performed with the following antibodies: estrogen receptor protein (ER) (dilution 1:100 DAKO), progesteron receptor protein (PR) (dilution1:100 DAKO), CA19.9 (dilution 1:50, BioGenex); cytokeratins (CK7, CK20) (dilution1:100, DAKO); gross cystic disease fluid protein 15 (GCDFP15) (dilution 1:100 Immunomarkers). All sections were controstained with Carazzi's hematoxylin. Histological examination of neoplastic tissue was consistent with atypical epithelial elements arranged in a single cell growth pattern, involved widely the entire stomach, also spreading through the whole thickness of the wall, from mucosa to perivisceral fat (Figures 1a and 1b).

Cells were monomorphic, with slight nuclear atypia and poor cytoplasm and sporadically intracytoplasmatic lumina were visible in few ones (Figure 1c). These architectural and cytological features can be typically seen in breast lobular carcinoma too. Immunohistochemistry showed reactivity for CK7 (Figures 1d and 1e), for ER (Figures 1f and 1g), PR and GDFP15 (Figure 1i), while CK 20 and CA 19.9 were negative.

**Figure 2:**
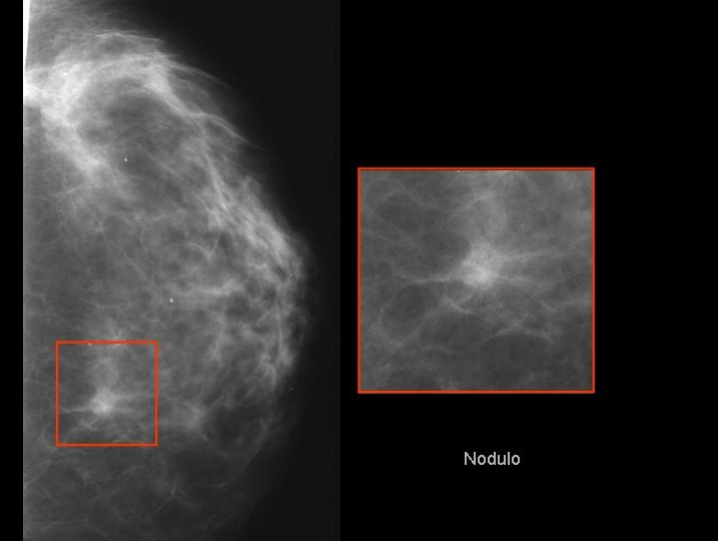
Mammography showed a nodular lesion with irregular margins of 1 cm in diameter, located in the lower outer quarter of the left breast.

**Figure 3:**
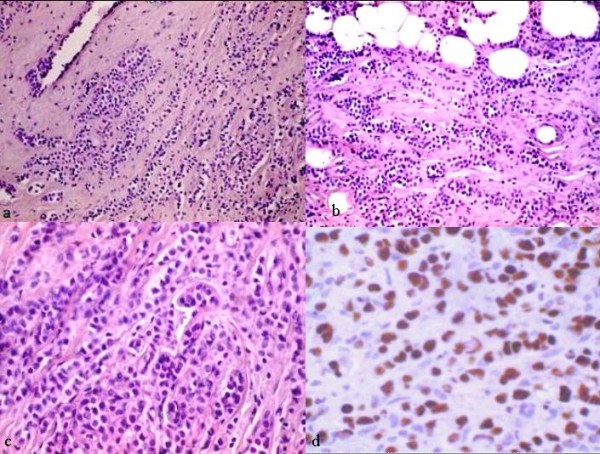
**Photomicorgraphs of breast**. a-b) Lobular carcinoma: small cells arranged in row and in single cells. (H&E, original magnification 20×). c) Lobular carcinoma: neoplastic cells with signet ring like appearance with univacuolated introcytoplasmatic lumina.(H&E, original magnification 40×). d) Lobular carcinoma: estrogen nuclear expression (original expression 40×).

It was evident that a complete histological and immunohistochemical analysis of the gastric specimens oriented now to a strongly suspected lesion as a metastasis arising from the breast. Therefore the patient was therefore contacted in order to investigate further. Mammography displayed a non-palpable lesion (max 1 cm in diameter) with irregular margins, located in the lower outer quarter of the left breast (Figure 2). Ultrasound examination confirmed that the lesion was possibly a cancer. Next the diagnostic stained tissue sections of surgical specimen demonstrated that it was, in fact, a lobular carcinoma of the breast (Figures 3a, b, c), with this immunoassaying profile ER + (figure 3d) (60%); PR + (40%); Ki67: 5%; human epidermal growth factor receptor 2 (Her-2) (DAKO) negative.

Postoperative hormone therapy was administered to the patient, who died, however, 10 months later.

## Discussion

Although the diagnosis of undifferentiated gastric carcinoma with neuroendocrine differentiation had been suggested from the microscopic observation (a scatter mucosal spread of neoplastic signet ring cells) of biopsy specimens, and on the basis of the poor clinical history, the diffuse and strong positivity for ER, PR, CK7 and GDFP15 as well as the negativity for CA 19.9 and CK20 suggested that the breast was the primary site of the neoplasm.

It is also a fact that the surgical examination of the breast demonstrated the presence of an impalpable mass consistent with an infiltrating lobular carcinoma, whose morphological (Figures 3a, b, c) and immunoistochemical characteristics of cells were almost identical to those of the stomach: ER+ (Figure 3d), CK7+/CK20-; GCDFP15+.

In the gastrointestinal tract it is of great value to distinguish a primary carcinoma from a metastatic one, in order to establish a suitable medical therapy in such patients, avoiding a surgical procedure. Linitis plastica originating from a metastatic lobular carcinoma of the breast is responsive to hormone therapy, to chemotherapy or both, particularly when metastases are positive to ER and PR. Nevertheless the prognosis is still poor with a median survival rate of two years following the diagnosis of gastric lesions [3].

Lobular breast cancer develops more frequently gastrointestinal metastasis than ductal carcinoma [6-10].

In 1980 Cormier et al. from the Mayo Clinic [11], first described linitis plastica as a metastatic lesion of an invasive lobular breast carcinoma. In the early stages metastases appear as a submucosal isolated lesion [2] producing a plaque-like or nodular or polypoide appearance [4] or otherwise irregular mucosal surface in the involved area, which in time, with a more extensive submucosal and muscular infiltration, looks macroscopically like a gastric carcinoma or lymphoma. Further because of blood dissemination of tumour cells, metastatic elements may diffusely involve all layers of the entire stomach, skipping or not the mucosa, resulting in a total lack of distensibility and in rigidity of the gastric wall such as in linitis plastica. These patterns are also characteristic of metastases from lobular carcinoma [12].

Interestingly in a model of spreading where neoplastic cells may often spare the mucosa, preoperative histological diagnosis can be very difficult, by reason of endoscopic biopsies are in many cases superficial and may lead to false negative results, that is endoscopic biopsy findings are normal in up to 50% of patients [13]. Furthermore the radiological appearance of linitis plastica from breast carcinoma metastases is quite similar to that of primary gastric cancer [12,13]. The barium swallow usually demonstrates mural rigidity, with thickening of the gastric wall.

CT detection of gastric metastases from breast cancer presents as widespread gastric wall thickening of more than 1 cm in an adequately distended stomach [14]. Recently Lorimier *et al*., [15] have reported that ultrasonography was effective for visualising linitis plastica in a small series of patients with gastric metastases secondary to breast cancer. Gastric cancer and breast metastasis share almost the same clinical, endoscopic and radiological features that do not help much in specifying whether the linitis plastica is primary or secondary.

Moreover, when also endoscopic biopsy was diagnostic, it is generally known that lobular carcinoma may also contain a large number of signet-ring cells that if combined with a gastric mucosal spreading pattern, can mean that the metastatic disease to the stomach once more is almost indistinguishable from primary gastric linitis plastica [16]. However it was remarked that breast SRCCs (Signet Ring Cell Carcinoma) might show some morphologic differences from gastric and colon SRCCs, [17]. In fact breast SRCCs might contain a single, well-circumscribed univacuolated intracytoplasmic lumina, with a central eosinophilic inclusion, whereas other SRCCs usually have the extended, globoid, and optically clear cytoplasmic acid mucin that pushes nuclei against the cell membrane On account of these differences might be difficult to detect in individual cases, and the morphologic similarity of various SRCCs on H&E-stained sections, immunohistochemical analysis has a key role in the determination of the tissue origins of metastatic SRCCs in spite of clinical history.

In this context the authors proposed an immunohistochemical algorithm, using successfully a panel of selected antibodies, CK 20, CK 7, ER, PR, and GCDFP15.

CK 20 proves to be particularly positive in gastric, colorectal, pancreatic and in transitional cell carcinomas, while it is not observed in any carcinomas of the breast [18,19]. CK 7 in contrast is extensively registered in 90% of carcinomas of the breast and its expression was also observed extensively in 50–64% of primary gastric adenocarcinomas [20,21]. For that reason CK 7 and CK 20 expression patterns, are very useful in metastatic lesions of uncertain origin. About 30% of gastric adenocarcinomas have the CK7+/CK20+ pattern; 20% are CK7-/CK20+, 10% have the CK7-/CK20- pattern and only 20% are CK7+/CK20 – [21-23].

Several studies have shown almost uniform negativity for ER in primary gastric carcinomas, Japanese authors have shown that up to 28% of these tumors may be positive, with a focal weak to moderate staining intensity [24-26].

Nevertheless the localisation and functionality of ER and PR receptors in tumoral gastric tissue remain unclear. Many authors have detected significant amounts of oestrogen receptor in normal gastric mucosa with lower amounts in cancer cells. For them this is consistent with steroid hormones having a protective action, and may contribute to the sex difference seen in the incidence of gastric cancer [27]. Recently, a new estrogenic receptor, called estrogen receptor beta (ER beta) [28], was found expressed in various tissues, including normal gastrointestinal tract. The expression of ER beta, in stomach adenocarcinomas has been investigated, specifically in signet ring cell adenocarcinomas, together with surrounding non-cancerous tissues. The effects of estrogen in stomach cancer, as well as those in normal stomach, may be mediated by ER beta so that the role of ER beta may differ by the subtype of stomach adenocarcinoma – specifically signet ring cell adenocarcinomas and other ones. Residual studies evaluated estrogen and progesterone receptors in gastrointestinal cancers, with conflicting results. They detected very low levels of receptors in normal and cancer tissues, suggesting a feature of the tissue rather than a consequence of a malignant process [29].

It's clear that the role of ER or PR in these cancers must still be elucidated such as if this unusual immunophenotype might cause a pitfall in gastric biopsy specimens. Furthermore cytoplasmic positivity for gross cystic disease fluid protein (GCDFP-15) may be also functional to confirm a mammary origin. Many reports have established that immunohistochemical detection of GCDFP-15 is a sensitive marker for lobular breast carcinoma and that it is a convenient addition in the diagnosis of metastatic carcinoma of suspected breast origin since that it has been found to be positive in breast cancers and negative in all primary stomach cancers. However GCDFP-15 has not been widely studied because of a 90% specificity for breast tissue, but a sensitivity of only 50% [30,31].

To recap mammary metastasis, as in our own case, may resemble primary GI carcinomas by radiologic, endoscopic, and, particularly, histological methods. So distinguishing between metastasis carcinomas of the breast and a primary gastric adenocarcinoma, especially poorly differentiated, diffuse or signet ring cell types, is a distinction without a difference, if based only on the morphology of both tumors.

Azzopardi [32] and then Battifora [33] in the past described a distinctive type of intracytoplasmic vacuole within tumour cells, characterized by the presence of a round globule of syalomucin that imparts a "target" appearance to the cell or by the presence of a single sharply demarcated intracytoplasmic vacuole, with or without a central eosinophilic inclusion, which was termed the "univacuolated lumen type" of signet ring cell. Battifora contrasted this with a second type of signet ring cell with "multivacuolated" cytoplasm, termed the "GI type" and proposed that the former type of cell may be specific for carcinoma of the breast. In our case, an almost prevalent component of univacuolated signet ring cells was observed. Therefore, in our opinion the morphologic appearance of the tumour cells in accordance of published criteria, was not of limited value in distinguishing metastatic invasive lobular carcinoma from primary gastric carcinomas.

Unfortunately, in many cases diffuse type gastric adenocarcinomas and lobular carcinomas of the breast often overlap their cytomorphologic features, showing a single-cell growth pattern and a mixture of types of signet-ring cells [16]. This fact suggests a more confident use of selected immunohistochemistry approaching to gastrointestinal adenocarcinomas, regardless of clinical or histological evidences, because primary and metastatic carcinomas of the GI tract have significantly different treatment and prognosis.

To perform this, we used a panel of antibodies, of different antigenic subtypes, that we believed might yield useful diagnostic information. These included the following ones that have traditionally been associated with breast carcinomas: estrogen receptor protein (ER), progesterone receptor protein (PR), gross cystic disease fluid protein (GCDFP15), and cytokeratins (CK7).

The reactivity for CK7 and GCDFP15, including hormone receptor expression, and for contrast, the negativity for CK20 and CA 19.9, were in this case of great value to differentiate an unsuspected lobular carcinoma from a gastric cancer.

Only after a correct diagnosis we were able to initiate the treatment targeted towards systemic breast cancer. Patients with linitis plastica from breast cancer metastases have been known to respond to hormone therapy or chemotherapy, or both, particularly if the metastases are strongly positive for oestrogen receptors. Surgery should be only reserved for palliation in cases of intestinal obstruction or bleeding. The prognosis of these patients is still uncertain. Generally gastric metastases reflect a poor prognosis [3]. In the series by Taal et al. the median survival from the time of diagnosis of gastric metastases was almost 2 years; only 6 (22%) of the 27 patients survived for more than 2 years [13]. On the other hand such a therapeutic approach is more likely to have a profound effect on survival especially if no other extensive metastases are present.

## Conclusion

We report a rare case of metastatic disease to the stomach arising from a non palpable lesion of the breast. Unlike previously reported cases, in which the primary breast lesion had been well recognised or was clinically evident, in this our case a breast cancer was found to be the primary tumour only after that gastrectomy had yet been performed, in a woman with no other pathological history than a "diagnosed" gastric cancer. Furthermore we describe a history report that can take away from the truth: an old female patient with a dyspeptic disorder and with no clinical signs of unhealthy breast; an esofagogastroduodenoscopy positive, which showed a vastly hyperaemic gastric mucosa, with nodular appearance of the fundus and corpus and hypertrophic plicae of antrum; a superficial biopsy with minimal tissue showing a mucosal spreading of diffuse monotonous neoplastic cells with signet-ring like appearance. Everything suggested the erroneous diagnosis of primary gastric adenocarcinoma.

To avoid a similar situations, we suggest an algorithmic use of targeted immunohistochemical markers in order to determine the primary site of gastrointestinal tumours. Making a primary gastric cancer appear different from a metastatic one, especially if it is of mammary origin, is a great challenge for a correct planning of the therapeutic approach, not only to act on survival but also to spare the patient unnecessary surgery. The Authors goal is to increase the awareness on this event among clinicians, pathologists and surgeons.

## Competing interests

The authors declare that they have no competing interests.

## Authors' contributions

AC and GT have made substantial contribution to conception and design, and in drafting the manuscript. GC has been involved in revising it critically for important intellectual content. AMM and GG has given final approval of the version to be published. LR has been involved in acquisition of data, analysis and interpretation of histopathologic dates and together with ED has been involved in interpretation of immunohisthochemistry data. All authors read and approved the final manuscript.
